# New Perspectives on Serialism and Parallelism in Oculomotor Control During Reading: The Multi-Constituent Unit Hypothesis

**DOI:** 10.3390/vision3040050

**Published:** 2019-09-25

**Authors:** Chuanli Zang

**Affiliations:** 1 School of Psychology, University of Central Lancashire, Preston, PR1 2HE, UK; czang@uclan.ac.uk; 2 Academy of Psychology and Behaviour, Faculty of Psychology, Tianjin Normal University, Tianjin 300074, China

**Keywords:** serialism, parallelism, oculomotor control, reading

## Abstract

Currently there are several computational models of eye movement control that provide a good account of oculomotor behavior during reading of English and other alphabetic languages. I will provide an overview of two dominant models: E-Z Reader and SWIFT, as well as a recently proposed model: OB1-Reader. I will evaluate a critical issue of controversy among models, namely, whether words are lexically processed serially or in parallel. I will then consider reading in Chinese, a character-based, unspaced language with ambiguous word boundaries. Finally, I will evaluate the concepts of serialism and parallelism of process central to these models, and how these models might function in relation to lexical processing that is operationalized over parafoveal multi-constituent units.

## 1. Introduction

Reading is a visually mediated psychological process. Humans process visual information via the eyes, and during reading, text is visually encoded and an abstract orthographic representation is formed. This abstract orthographic representation is then used to undertake lexical processing whereby a word’s syntactic category and meaning are accessed. The physiological basis of the human retina is important in relation to reading. At (approximately) the middle of the retina, the fovea, a small circular region (roughly two degrees across) where visual acuity is the highest, delivers detailed visual information about the environment [1]. Thus, readers visually perceive a small detailed region of text immediately around the point at which they are directly fixating, while beyond this, in the parafovea (extending to five degrees on each side of fixation), the text is visually degraded. In order to obtain clear information for comprehension, readers have to make a series of saccades and move their eyes frequently to place the point of fixation on the upcoming text. When a reader fixates a word, they not only process that word itself, but also the upcoming words from parafovea [2,3]. Readers spend less time fixating a word when it is parafoveally available compared to when it is masked or removed [4]. This advantage is referred to as the *preview benefit* [2] and indicates that partial information about parafoveal words is available prior to their direct fixation. It is well known that during reading the oculomotor control system makes two moment-to-moment decisions: one temporal—when to move the eyes, and one spatial—where to move next. The “when” decision refers to how long it takes to fixate a word, captured by fixation duration measures. The “where” decision (in relation to progressive saccades) refers to which word is selected as the upcoming saccadic target, and the specific position where the eyes actually land on a target word, captured by fixation probability and fixation location measures. A number of researchers have endeavored to explain how the two decisions occur by developing various types of computational models of eye movement control during reading.

It is widely accepted that eye movements are under cognitive control during reading [5,6,7,8], and are both central to the process of reading, and constrain the rate at which orthographic information is encoded and processed by the written comprehension system [9]. Currently, there are several cognitive computational models of eye movement control that provide a good account of oculomotor behavior during reading of English and other alphabetic languages. I will begin by briefly providing an overview of two dominant cognitive models that are currently used to guide reading research: E-Z Reader [10,11,12] and SWIFT [13,14,15], as well as a model that has been recently proposed: OB1-Reader [16]. I will then evaluate a critical issue of controversy among these models regarding whether words are lexically processed serially or in parallel, and consider reading in Chinese, a character-based, unspaced language with ambiguous word boundaries. Finally, a hypothesis concerning processing of multi-constituent units will be proposed as a potential solution to the current serialism/parallelism impasse.

## 2. An Overview of Models of Oculomotor Control during Reading: Serial or Parallel?

### 2.1. E-Z Reader Model

E-Z Reader [10,11,12] is the most elaborate of sequential attention shift (SAS) models during reading which share the two basic assumptions: (1) attention acts like a spotlight and focuses on only one word at a time; (2) attentional shifts occur sequentially from one word to the next, in order to keep serial word order for comprehension. In this model lexical identification is the engine moving the eyes forward during reading. Specifically, lexical identification is a two-stage process: the early stage of processing, *L*_1_, corresponds to a *familiarity check*, an assessment of the familiarity of the upcoming word based on its frequency of occurrence and predictability from the preceding context in a sentence. *L*_1_ is modulated by visual acuity (determined by the mean absolute distance between the current fixation location and each letter in the word being processed [17]) such that long words and/or words located further away from the center of the fovea are processed less efficiently and are thus fixated for longer than short words and/or words located closer to the center of the fovea. E-Z Reader posits that the completion of *L*_1_ causes the oculomotor system to start programming a saccade to the next word. The later stage of processing, *L*_2_, corresponds to *lexical access*. The completion of *L*_2_ causes attention to shift from the currently fixated word (the word that has been lexically identified) to the next parafoveal word, after which parafoveal processing of that word can occur (though note that the eyes remain on the currently fixated, foveal word, as the attentional shift is fast and usually happens before the eyes actually move to directly fixate the parafoveal word [18]). To reiterate, lexical processing of the next parafoveal word only starts after lexical processing of the currently fixated word has been completed.

With respect to saccade targeting, E-Z Reader presumes that the oculomotor system uses low-spatial frequency information such as word boundaries (indicated by interword spaces in English and most other alphabetic languages) to select the next, unidentified, parafoveal word (by default) as a saccadic target. Furthermore, saccades are often targeted towards the center of a word—the best place to fixate a word in order to recognize it most efficiently ([19,20], though see also references [21,22]). Due to several factors including systematic bias and random motor errors, saccades do not land precisely at the word center but actually at a position slightly to the left of the center of a word, the preferred viewing location (PVL, [23]). If an upcoming word is not selected as a target, then it is skipped. E-Z Reader assumes that if the *L*_1_ stage of lexical processing of the parafoveal word is completed very rapidly, then prior to the completion of programming of the saccade from the foveal word to the parafoveal word, a new saccade to the word beyond the adjacent parafoveal word will be generated and the parafoveal word will be skipped.

According to E-Z Reader, lexical processing drives the eyes to move from one word to the next and saccade targeting is made on a word by word basis, with upcoming words being selected as the next saccadic target. E-Z Reader provides an account for a wide range of findings including effects of word length, frequency, predictability, parafoveal preview, foveal load with parafoveal preview, spillover, skipping cost (fixations are longer prior to skipping words than fixating words), post lexical processing and so on [10]. However, critically, in the framework of E-Z Reader, both foveal and parafoveal processing occur in a serial manner mediated by a process of sequential attention allocation, and they do not take place simultaneously. In other words, more than one word cannot be processed lexically at a time. On the basis of these assumptions, according to this view, lexical or sub-lexical properties of the parafoveal word do not have a direct influence on processing of the currently fixated, foveal word. Thus no so-called lexical parafoveal-on-foveal effects are expected (e.g., reference [24]).

### 2.2. SWIFT Model

The SWIFT model [13,14,15] is the most developed of the parallel processing gradient (PG) models during reading. It assumes that attention is spatially distributed across an attentional gradient spreading over multiple words to support parallel lexical processing. PG models are based on the dynamic field theory of movement planning such that a field of activation is spatially distributed over several potential movement targets and the spatially-distributed pattern of activation determines the probability of selecting a saccade target in the activation field. In the SWIFT model, each word of a sentence indicated by spaces is the unit of the activation field that changes over time due to word identification. As soon as words fall within the activation field, they start to accumulate activation. Activation is built up in the first stage of preprocessing, decreases in a second stage of lexical completion, and tends to zero after a word is completely identified. The relative activation associated with lexical identification determines the probability of which word is selected as a saccade target, therefore saccade target selection is a competitive process among all of the activated words within the attention gradient (the span of effective vision), and the one with highest activation is most likely to be selected as the next target. 

Note, in a recent version of the SWIFT model [25], a dynamically modulated processing span was incorporated such that attentional deployment might vary in size from a sharp, narrow focus to a widely broad area (i.e., a zoom lens of attention) and this is modulated by processing difficulty of the fixated word. If the foveal word is difficult (e.g., a low frequency word) and its activation is increased, this causes the processing span to be narrow and probably only the fixated word to be processed during a fixation. However, if the foveal word is easy (e.g., a high frequency word) and its activation is reduced, this causes the processing span to be dynamically increased and extend over a number of words. In terms of when to move the eyes, SWIFT assumes that the decision to program a new saccade is generated by a random timer, but an inhibitory control process, foveal inhibition, modulates the progress of the random timer and the processing time, via the difficulty of the currently fixated word. Lexical processing rate is constrained by visual acuity, with increased processing speed for words closer to the point of foveal region. Therefore, it is likely that foveal words are identified more rapidly than parafoveal words. However, the lexical activation level of a word is related to its processing difficulty constrained by its frequency. Thus, when the eyes are fixating the foveal word *n*, if word *n* + 1 is a high frequency word but word *n* + 2 is a low frequency word, then word *n* + 2 might likely have a higher level of lexical activation compared to word *n* + 1. As a consequence, word *n* + 2 will become the next saccade target. Similarly, if word *n* − 1 has been previously fixated or skipped but has not yet been fully recognized, then it might have a higher level of lexical activation, resulting in a regression to word *n* − 1. 

SWIFT captures many patterns of eye movements and provides accounts for reading related phenomena including effects of word length, frequency, predictability, skipping cost and benefit (fixations are shorter prior to skipping short and/or high frequency words) and regressions. Critically, SWIFT assumes that multiple words within the perceptual span [26] can be lexically identified in parallel. This claim of parallel processing is supported by observations of (1) parafoveal-on-foveal effects mentioned earlier whereby processing of the parafoveal word *n* + 1 influences fixation durations on the currently fixated foveal word *n*; and (2) word *n* + 2 effects whereby there is an observable influence of the lexical properties of words two to the right of the currently fixated word *n*. However, there have been questions as to whether both types of effect are reliable. Parafoveal-on-foveal effects are relatively weak and the observation of them is mainly restricted to corpus studies as opposed to carefully controlled experiments that require reading for comprehension [24,27,28]. And word *n* + 2 effects are subtle and most often reported when word *n* + 1 is a short or high frequency word [17,29,30]. Furthermore, the SWIFT model has received criticism in relation to how the attention gradient mechanism might account for effects associated with comprehension difficulty should words be identified out of order, and how readers might maintain word order to support incremental interpretation when this occurs [31].

### 2.3. OB1- Reader

Recently, Snell et al. [16] sought to integrate ideas associated with models of visual word recognition and those with eye movement control in reading. They proposed a computational model of reading called the OB1-Reader. In line with the SWIFT model, OB1 adopts the approach of parallel graded attention (PG) supporting the position that multiple words can be identified in parallel within an attentional visual input window (comprising five words—the fixated word *n*, along with words *n* − 2, *n* − 1, *n* + 1, *n* + 2). Furthermore, OB1 adopts the approach of relative letter position coding for word recognition to support parallel processing at the letter level. OB1 assumes that visual input activates nodes that represent the relative position of letter pairs of a word (open bigram nodes). For example, the visual input *word* can activate nodes for *wo*, *wr*, *wd*, *or*, *od*, or *rd*, these nodes (e.g., *wo*) in turn activate all related lexical representations (e.g., *word*, *work*, *world*, *wonder,* etc.). The activation of letters in the visual input is constrained by visual acuity, attention, and crowding, with stronger activation of letters that are closer to the fixation and spatial attention, and weaker activation of letters that are crowded by other letters (central letters receiving more benefit from acuity, but outer letters receiving more benefit from reduced crowding, [32]). The open-bigram nodes subsequently activate word nodes via bigram-to-word excitation (activating bigram nodes that are part of the word node) and word-to-word inhibition (inhibiting word nodes that share the same bigrams). The activation of a word node is determined by its length, frequency, and predictability from the preceding context in a sentence. When its activation reaches a recognition threshold, it is identified. Also, if there is orthographic overlap between parafoveal and foveal words, then parafoveal information has a facilitatory influence on the word representation associated with the foveal word, resulting in an orthographic parafoveal-on-foveal effect [33].

Recall earlier, in the context of the parallel processing framework such as SWIFT, if words are identified out of order, the question has been raised as to how readers are able to keep track of the correct sequential order of the words, arguably, necessary for a veridical interpretation of sentential meaning [31]. In order to handle this issue, OB1 posits that all activated words are mapped onto a spatial location in a spatiotopic sentence-level representation, based on expectations about the number of to-be-recognized words, as well as the approximate length of each word indicated by interword spaces in the visual input. If the activation of a word reaches its recognition threshold, the length of the activated lexical representation must match with the length of an expected word at a location in the sentence-level representation in order for identification to occur. Note that these expectations can only be generated when spaces between words demarcate word boundaries (and therefore word lengths). The sentence-level representation is also constrained by syntactic structure. If word *n* is an article, then there may be an increased expectation for word *n* + 1 to be a noun or an adjective [33]. When a word is identified, attention moves forward to the next most salient location. The visual salience of each word in the visual input is modulated by visual acuity with which its constituent letters are processed relative to the point of fixation. Words that are closer to the point of fixation are more salient, and more likely to be selected as saccade targets. Therefore, in terms of the saccade targeting, OB1 operates similarly to the SWIFT model. 

OB1 can account for a range of effects such as word length, frequency, predictability, neighborhood size and orthographic parafoveal-on-foveal effects. However, it cannot explain lexical parafoveal-on-foveal effects even though it adopts the framework of parallel processing in reading. According to OB1, a high frequency parafoveal word *n* + 1 increases rather than decreases fixation durations on word *n*, as the activation of nodes associated with a high frequency word *n* + 1 exerts increased inhibition of nodes associated with word *n*. This is a prediction opposite to that of the SWIFT model. Furthermore, one of the theoretical advances of OB1 is considered to be the use of spatiotopic representations guided by word length. However, it is not clear how readers might generate differential expectations for words based on length if, for example, a sentence is comprised of words of equal length (e.g., *That tall girl must want some food*, see references [21,22]). Though, recently, Mirault, Snell and Grainger examined reading without word spaces, and argued that readers might need to identify the currently fixated word and use its word length information to estimate the left boundary of the next word, then make a saccade beyond that boundary. In this case, readers might engage in a more serial word-by-word identification strategy rather than the parallel process that occurs during spaced text reading. The question arises regarding how such word identification processes might operate in the absence of word boundaries in reading unspaced text [34]. Finally, when word length information is not immediately obvious in reading unspaced languages such as Chinese, a language in which it has been shown that the length of a word influences word identification and saccadic targeting [35], it is an interesting challenge for OB1 to explain how readers segment the text into words in order to use word length information to keep track of word order and maintain a sentence-level representation.

## 3. The Current Challenge: The Concept of a Word and Its Role in Chinese Reading 

A critical issue of controversy and conflict among the three models concerns whether words are lexically processed serially or in parallel during sentence reading. According to E-Z Reader, lexical processing occurs in a serial manner whereby words are lexically processed sequentially one at a time. In contrast, SWIFT proposes that multiple words around the point of fixation (within the perceptual span) are lexically processed in parallel, and therefore, potentially out of sequential order. OB1 adopts the approach of the SWIFT model stipulating parallel processing of multiple words, and also integrates the approach of relative letter position coding to support parallel letter identification in multiple letter strings. In order for the parallel processing system to identify multiple words at a time without misperceiving word order in reading, OB1 constructs a spatiotopic sentence-level representation and uses visual word length information to generate expectations regarding which activated word representation belongs to which spatial location in working memory. In these accounts fixations and saccades are considered to fundamentally constrain the delivery of visual information to the brain for linguistic processing, and therefore, are formative with respect to the nature of such processing. Thus, in the context of these competing models, these theoretical issues (serialism vs. parallelism; sequential vs. non-sequential lexical processing; formation of spatial mappings etc.) are extremely important in relation to eye movement control during reading, because it is widely accepted that word identification is a primary determinant of when a reader makes the decision to move their eyes from the current word to the next point of fixation. 

It should be clear that the current debate among these competing models has been mainly limited to the reading of alphabetic languages like English, German, and French. Written word identification in these languages seems relatively straightforward, as it involves words that are comprised of adjacent letters, have pronunciation and meaning, and are visually separated from other words in a sentence by spaces [31]. However, this is not the case in a number of alphabetic scripts. For example, there are no spaces to define words in Thai [36], and there is often ambiguity as word segmentation in Thai relies heavily on sentential context [37]. Even in a spaced language like Finnish, a highly agglutinative language, a word might be comprised of multiple constituent sub-words that appear together without spacing. For example, “lumi” is the basic form of “snow”, “lumipallo” means “snowball”, “lumipallosota” means “snowball fight”, and “lumipallosotatantere” means “snow ball fight field” [9,38]. It is likely that Finnish readers segment and encode the orthography of such words in chunks or units smaller than the entire word, though it remains unclear as to exactly how they determine the units of orthography to encode during a fixation [38,39,40]. Also in English, the same compound can be written as a single word such as “lifestyle”, a hyphenated word that may be processed as a single word or two words such as “life-style”, or two separate words such as “life style” [41]. It should be clear that it is challenging to consider what actual form a lexical unit takes in reading of alphabetic languages, and whether the concept of a word in the process of word identification is constant across different language scripts. 

The issue becomes more complex when taking into account a non-alphabetic language with completely different orthography. Chinese is logographic and character based, being formed of closely packed box like characters that comprise sentences. Characters are formed from strokes with different visual complexity but occupying the same area of space. Words are comprised of one or more characters. However, word units in Chinese are not clearly demarcated by spaces at their beginning and end. There are no visual cues or inflectional indicators (e.g., lexical categories, number demarcations, tense demarcations, etc.) to mark words’ syntactic properties. In fact, the Chinese did not have a term for “word” until the concept was imported from the West at the beginning of the 20th century [42]. It is, perhaps, not so surprising therefore, that Chinese readers sometimes do not have a clear concept of what a word is in Chinese, and often do not discriminate words from other linguistic units like phrases [43,44,45,46,47]. In word segmentation tasks where participants are required to put a “/” between the words of a sentence, different native Chinese readers often segment the same sentence into different word units, and they frequently demarcate strings of characters comprising several words as a single word [46,48]. Despite the ambiguity regarding word boundaries in Chinese, there is considerable evidence suggesting that the word has psychological reality during Chinese reading (note, in this line of research the word unit is most often defined according to the dictionary definition, also any strings with ambiguous word status are precluded from experiments following prescreening procedures, see reference [45] for a review). For example, characters that belong to a word are processed efficiently as a whole unit (e.g., reference [49]). If spaces are inserted between the constituent characters of a word, reading is slowed [43]. By contrast, if spaces are artificially introduced between words, reading is facilitated for children [50,51] and learners of Chinese as a second language [52]. Furthermore, it has been demonstrated that Chinese readers use statistical lexicality cues (i.e., some characters are more likely to be a single character word—single character word likelihood [53]; and some characters are more likely to appear at the beginning or end of a word—within word character positional frequency [54,55,56]) to facilitate word segmentation processes during reading. 

To this extent, word segmentation and the nature of lexical processing are tightly intertwined in Chinese reading. In order to lexically identify a written word in a Chinese sentence, it is necessary to make decisions about which characters are constituents of the word, and which are not. That is, it is essential to segment the characters that comprise the word in relation to those in the sentence around it. Note again, there are no spaces to demarcate words in Chinese text. Currently, not only do the E-Z Reader, SWIFT, and OB1 models offer conflicting accounts of the nature of lexical processing that occurs across fixations during reading, but they also have no mechanism for word segmentation within them. This represents a significant limitation with respect to the generality of these models to unspaced languages.

There has been some effort to explore how words may be segmented during Chinese reading. Li, Rayner, and Cave [57] proposed an account of word segmentation on the basis of the interactive activation framework [58]. They assumed that Chinese characters within the perceptual span are processed in parallel, with processing of each character being constrained by visual acuity and attention. Activation of representations at the character-level passes to corresponding word unit representations at the word-level. Subsequently, activation from the word units passes back to the constituent character representations, and then forward once more through the system. Characters that are part of a word are activated faster and to a greater degree compared to the other characters. The word-level representations compete with each other until the most activated word unit wins the competition, at which point the word is recognized and it is automatically segmented from the character string within which it is embedded. Thus, in Li et al.’s account, word segmentation and lexical identification are part of a single unified process. However, this model currently only accounts for lexical identification of four-character units, and offers no account of lexical identification in relation to saccadic targeting during normal sentence reading. Until now, no model that specifically focuses on eye movement control during Chinese reading has been put forward, though Rayner, Li, and Pollatsek [59] examined whether E-Z Reader could be extended to Chinese reading. Their work did suggest that the model may offer a potential account, though the centrality of the word unit and its identification to oculomotor decisions in this framework meant that the issue of word segmentation remained a critical (unexplained) aspect of processing. It seems fair to suggest that at present it is far from clear how a sequence of characters are segmented into words and how attention is allocated and processing operationalized across those characters, or words, or phrases, in unspaced languages like Chinese.

## 4. Beyond Serialism and Parallelism: A Multi-Constituent Unit Hypothesis 

The characteristics of the Chinese writing system provide challenges for the debate over serialism versus parallelism in oculomotor control during reading. As mentioned earlier, whilst a significant amount of eye movement research has suggested that words are processed serially [31], it is also the case that an increasing number of studies have shown effects that indicate, under certain circumstances, words may be processed in parallel [28]. For example, when word *n* + 1 is a short or very high frequency word, or when word *n* + 1 is grouped with its spatially adjacent word(s) to form a single unit (as will be discussed below), readers are able to obtain useful linguistic information from word *n* + 2 while fixating word *n* and parafoveal processing can extend across more than two spatially adjacent words. Also semantic information about the parafoveal word *n* + 1, under some circumstances (e.g., for synonyms), can be acquired sometimes while fixating the foveal word [17]. Such effects occur particularly in Chinese reading [60,61]. If one adopts a traditional word-based processing approach, then such evidence would be favorable for parallel models and problematic for serial models. Note though, as mentioned earlier, there has been some effort with SWIFT to show that the attentional window can be dynamically modulated by the properties of the fixated word (the zoom lens model of attention), and on this basis the model can be made to behave in a more serial-like manner if foveal load is high, but a more parallel-like manner if foveal load is low. This seems to be a comparatively flexible position with regards to a stipulation of serialism or parallelism of lexical processing. However, further research is needed to investigate the extent that foveal load has an influence on the depth and/or spatial extent of parafoveal processing [62]. Currently, it is reasonable to say that we have reached something of an impasse in the serial/parallel debate, and this situation necessitates some rethinking in relation to the central concepts of the debate (i.e., serialism and parallelism) and how models based on these ideas might function. Specifically, I will consider how lexical processing may be operationalized over a more flexible unit of text than the word.

In an attempt to move the issue forward, a theoretical hypothesis, the Multi- Constituent Unit (MCU) Hypothesis has been developed that may, at least in certain circumstances, offer a way of reconciling parallel processing of words during reading with serial accounts. The account was put forward in a keynote address to the European Conference on Eye Movements by Liversedge [63] and has been outlined briefly in published work [64,65]. The idea behind the MCU Hypothesis is that some linguistic units that occur in the language, are comprised of multiple words, such as spaced compound words, binomial word pairs, idioms, and other common phrases, and these may be represented lexically as single representations. To be clear, parallel processing of multiple individual words (that are not MCUs) remains entirely consistent with a parallel account (e.g., SWIFT), and fundamentally problematic for advocates of the serial processing account (e.g., E-Z Reader). However, if words that are processed in parallel comprise a MCU, and MCUs are represented and stored in the lexicon as a single lexical entry, then in fact any demonstration of parallelism over the constituent elements of a MCU need not violate serial processing assumptions. This is the central theoretical claim of the MCU Hypothesis.

Some empirical evidence has shown that spaced compounds operate as MCUs in English [64], and that foveal processing can occur across MCUs in Chinese [65]. Cutter et al. [64] constructed sentences containing spaced compounds, that is, MCUs comprised of two frequently co-occurring words that refer to a single concept (e.g., *teddy bear*). They employed the boundary paradigm [2] and positioned the invisible boundary before the first constituent of a spaced compound (e.g., *teddy* in the sentence “*The small child gently cuddled his fluffy teddy bear while trying to get to sleep*”). In this paradigm, prior to the eyes crossing the boundary and directly fixating the target word, a parafoveal preview is presented to the reader. When readers’ eyes cross the boundary, the preview is changed to the target word and reading times on the target provide an index of the degree to which the preview is processed before it is actually fixated. In Cutter et al.’s experiment, they orthogonally manipulated the preview of each constituent to be either an identity preview, or a nonword, to examine whether preview benefit could be observed for each constituent (e.g., word *n* + 1 ‘*teddy*’ and *n* + 2 ‘*bear*’) while readers were currently fixating the preceding word *n* (e.g., ‘*fluffy*’). The whole of the spaced compound was displayed correctly when a saccade crossed the boundary. They found an interaction between the previews of each word, such that there was an *n* + 2 preview benefit only when there was an identical preview of word *n* + 1. In other words, processing of the second constituent of the spaced compound occurred only if this was “licensed” by the presence of the first constituent (note, as mentioned earlier, that *n* + 2 preview effect has only been reported when word *n* + 1 is short and highly frequent, and that E-Z Reader normally is not ready to explain *n* + 2 effects when word *n* + 1 is relatively long and of relatively low frequency making it unlikely to be skipped as per the Cutter et al. experiment). Cutter et al. argued that these results are due to the two constituent words being represented as a single unified lexical entry, a MCU, and that lexical identification of the whole MCU occurred directly. They also argued that the results are consistent with the view that MCUs are psychologically real and are represented lexically.

The Cutter et al. study has important implications for computational models of eye movement control in reading. As per E-Z Reader, processing operates serially with lexical processing being operationalized sequentially over each adjacent lexical unit within a sentence. Most often such units are individual words, but of course, some lexical units are MCUs that have a single lexical representation (corresponding to the entire MCU). Note also that lexical identification operates in the standard way according to E-Z Reader based on the parafoveal familiarity of the MCU. Thus, within the E-Z Reader framework, it is possible to maintain the central tenet of seriality that this model relies upon, whilst also readily explaining how “parallel” processing of some words (MCUs) might occur. In this way, the MCU Hypothesis offers a step change in theoretical thinking, moving us beyond the current serialism/parallelism impasse. 

Next, let us consider Chinese reading. To date, there has been some preliminary research investigating whether the lexical status of multi-character strings (i.e., whether they are processed as a single word or two separate words) exerted an influence on how they were processed. For example, Cui et al. [66] used the boundary paradigm and manipulated the preview of the second constituent (an identity character, or a nonsense character) of a monomorphemic word, a compound word, or a phrase. They found increased fixation durations on the first constituent when the preview of the second constituent was a nonsense character rather than when it was the character itself—a reliable parafoveal-on-foveal effect, and this effect only occurred when the first constituent was part of a monomorphemic word, but not when it was part of a compound word or a phrase (for similar findings in English reading, see [67]). Also, preview benefit on the second constituent was numerically larger for monomorphemic words than compound words and phrases, but comparable between the latter two. Apparently, the linguistic category of the character string modulates parafoveal processing in Chinese reading. Presumably, due to morphological or probabilistic characteristics of monomorphemic words, the first constituent licenses preprocessing of the second constituent since the two together form a meaningful unit, and thus, they were processed in parallel. In contrast, when the characters formed a compound word or a phrase, no such licensing occurred and the two constituents were processed serially. This argument was confirmed in an off-line assessment of judgements regarding the lexical status of the target character strings for phrases and compounds in the sentential contexts. 74% of compounds were rated as words (MCUs), whereas only 45% of phrases were rated as words (MCUs). The ambiguity with regards to the concept of a word or a phrase and the very low frequency of the compound word (3 per million) in this experiment might result in the two constituents of the compound word being processed serially.

In a follow-up study, Drieghe et al. [68] examined parafoveal processing of the second constituent of an adjective-noun or a noun-noun Chinese compound to investigate whether the morphological structure of Chinese character strings affected how they were processed. They observed a larger preview effect for the adjective-noun compound than for the noun-noun compound (for similar findings in English reading, see reference [69]). They argued that for the noun-noun compound, each constituent contributes similarly to lexical identification, whereas for the adjective-noun compound, the adjective modifies the noun, and the noun plays a greater role in lexical identification. It is clear that the lexical characteristics of compounds influenced parafoveal processing in Chinese reading. That is to say, how the compound was represented lexically directly influenced how processing associated with the identification of the compound was operationalized over its constituents when they were in the parafovea. This is a significant point in relation to the MCU Hypothesis. 

Beyond the investigation of two-character compounds and phrases, Yu et al. [65] examined whether there are linguistically meaningful lexical representations associated with idioms that are comprised of three characters (a single-character verb and a two-character noun). Yu et al. obtained evidence to suggest that the idioms were processed foveally as a single lexical unit. More recently, with more careful and systematic stimulus generation, we obtained strong evidence that some idioms with a modifier-noun structure are processed both parafoveally and foveally as MCUs [70]. Overall, these studies provide evidence that the unit over which parafoveal and foveal processing is operationalized in reading should be considered more flexibly. Indeed, the fact that Chinese is dense, character based, unspaced and extensively ambiguous in relation to word boundaries, provides an optimal written language situation in which to study how real time word identification and oculomotor processing occur over stimuli with indefinite lexical status during natural reading. 

It is important to note that an existing body of work on theories of alphabetic language use and processing is directly relevant to the MCU hypothesis, and the current proposal grows from this research. Earlier studies have provided explanations of why our mental lexicon might store multiple word units. For example, the usage-based theory [71] posits that the representations associated with a language are based on experiences with it. When a particular word sequence is encountered frequently, it gradually comes to be processed as a single unit, since frequent exposure strengthens its representation in memory, making it easier to be accessed as a whole. Similarly, the exemplar-based theory, often referred to as data-oriented parsing [72], contends that there is “universal representation” (rather than universal grammar) in language cognition, and assignment of the representation during language acquisition and processing relies solely on statistics. That is, the probability of a word sequence being represented as a certain constituent is computed entirely from linguistic experience. Therefore, both theories presume that linguistic units including words, as well as phrases, are represented and processed similarly, and thus are comparably affected by the frequency of occurrence. To be clear, frequently occurring multiple word units can be lexicalized alongside individual words as individual units in the lexicon. Even in the Words-and-Rules theory wherein there is a distinction between the lexicon and the grammar [73], it has been proposed that some memorized chunks (sometimes called listemes) that are larger than a word such as idioms and collocations, and that cannot be generated according to rules but should be lexicalized and processed as wholes. In line with these models, an increasing amount of evidence has indicated that our mental lexicon not only contains representations of individual words, but also frequently occurring multi-word units (so-called formulaic sequences, see references [74,75,76]) including collocations, idioms, binomials and lexical bundles, because formulaic sequences are processed more quickly and easily than matched non-formulaic phrases [74,75,76,77,78]. Some analyses show that at least 30%-50% of the written and spoken discourse is comprised of formulaic sequences [79,80]. The widespread existence of MCUs in language provides an excellent opportunity to establish what units can, and are, lexicalized, and what the criteria are for lexicalization during reading. The lack of distinct boundaries within multi-word sequences suggests that a flexible (perhaps more probabilistic rather than categorical) view of the processing unit should be incorporated in models of eye movement control during reading (for more discussions regarding the existing probabilistic models of morphological processing and sentence comprehension that may indirectly implicate multiword sequences, see reference [77]). Of course, much more empirical work is necessary to evaluate the MCU Hypothesis in natural reading of alphabetic languages like English, as well as non-alphabetic languages like Chinese.

## 5. Conclusions 

In summary, the current models of eye movement control have provided a substantial understanding of the cognitive and oculomotor processes involved in reading of most alphabetic languages such as English, German, and French, and they have motivated a large amount of experimental research on eye movements and reading. These models have different assumptions with respect to the nature of attention allocation, lexical processing and saccade targeting. Regardless of their differing stipulations as to whether words are processed serially or in parallel, they state that word identification fundamentally determines when to move the eyes and can also affect where to move next. In other words, both these decisions in relation to oculomotor commitments in reading are considered to be word based, and the concept of a word has been considered to be rather constant, static and fixed across scripts. However, as suggested here, this may not always be the case, particularly when we consider reading of non-alphabetic, unspaced languages.
